# Supplementary material for: An avian cortical circuit for chunking tutor song syllables into simple vocal-motor units
Source: Nat Commun. 2020 Oct 6;11:5029. doi: 10.1038/s41467-020-18732-x (PMC7538968; doi:10.1038/s41467-020-18732-x)
Supplement: Supplementary file 1 — Supplementary Information [file 41467_2020_18732_MOESM1_ESM.pdf]

Supplementary Figures

An avian cortical circuit for chunking tutor song syllables into simple vocal-motor units

Mackevicius et al.

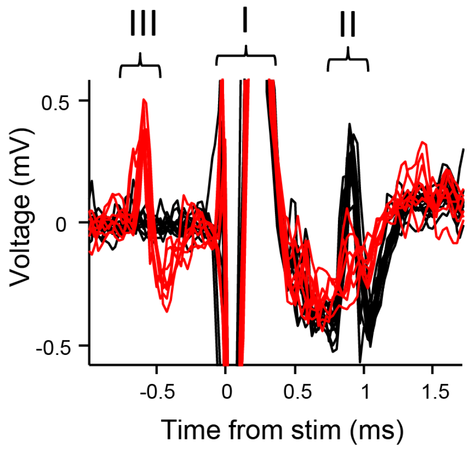

Figure S1: Example collision test of $\mathrm{NIf_{HVC}}$ neuron

(I) Artifact from stimulation in HVC, (II) antidromically identified spikes recorded in NIf. HVC-projecting NIf neurons ($\mathrm{NIf_{HVC}}$ neurons) fire antidromically-evoked spikes with low ($< 100\mu s$) jitter. When antidromic stimulation is triggered after a spontaneous $\mathrm{NIf_{HVC}}$ neuron spike (III, red traces), the antidromic spike collides with the spontaneous spike (missing spike at III, red traces).

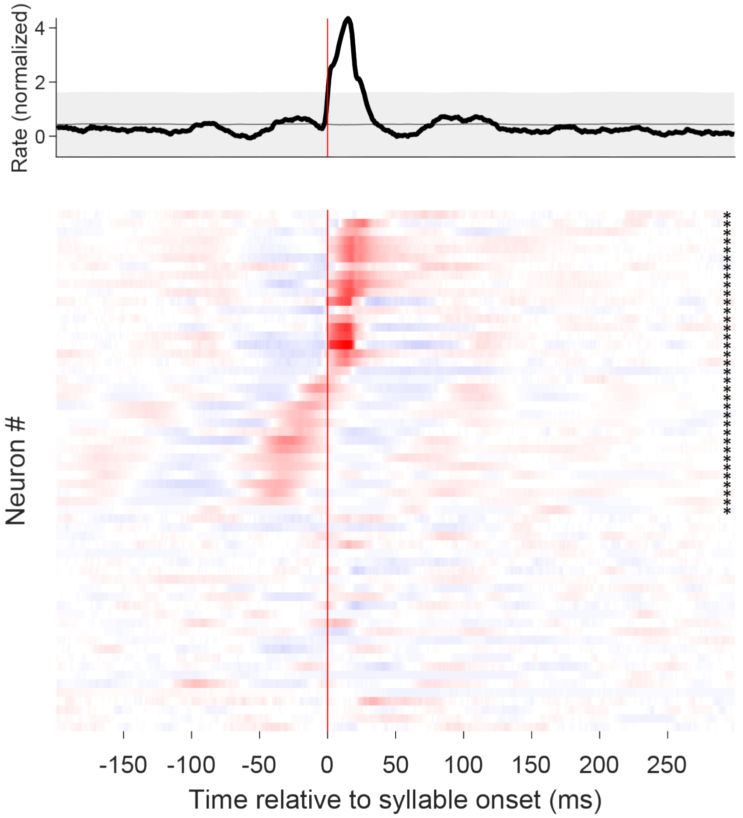

Figure S2: Song-locked activity during singing for neurons that are not identified NIf_{HVC} neurons

Summary of all syllable-locked activity recorded during singing for neurons that are not identified NIf_{HVC} neurons. Each row corresponds to an onset-aligned PSTH from a different neuron. PSTHs are normalized by subtracting the mean and dividing by the standard deviation of the PSTH; red indicates values above the mean, blue below the mean. Significant neurons (asterisk at right) are sorted by the latency of their peak firing rate. The upper plot shows the mean normalized firing rate across all of these neurons.

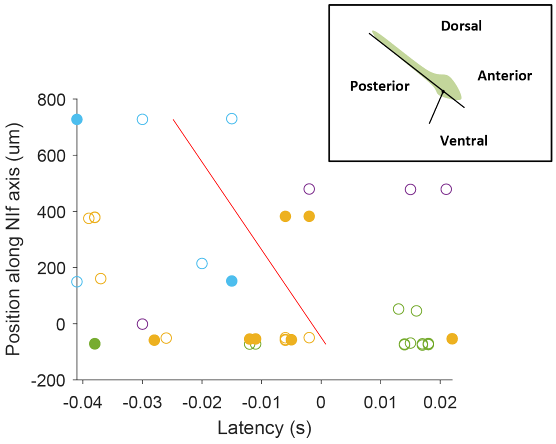

Figure S3: Anatomical position of neurons with different response latencies
As a function of latency, the estimated anatomical location of each recording site along the long axis of NIf. Zero corresponds to the mesopallial lamina. Inset shows a schematic of a sagittal view of NIf relative to the mesopallial lamina. Different colors represent different birds. Filled circles correspond to HVC-projectors. The data included in the figure are the cases where: (1) it was possible to unambiguously estimate in the histology which track corresponded to which recording electrode, and (2) the song-locked PSTH had a significant peak, so it was possible to estimate the neuron's response latency (33 neurons, including 11 projectors from 6 birds). The R^2 value for these points is 0.15.

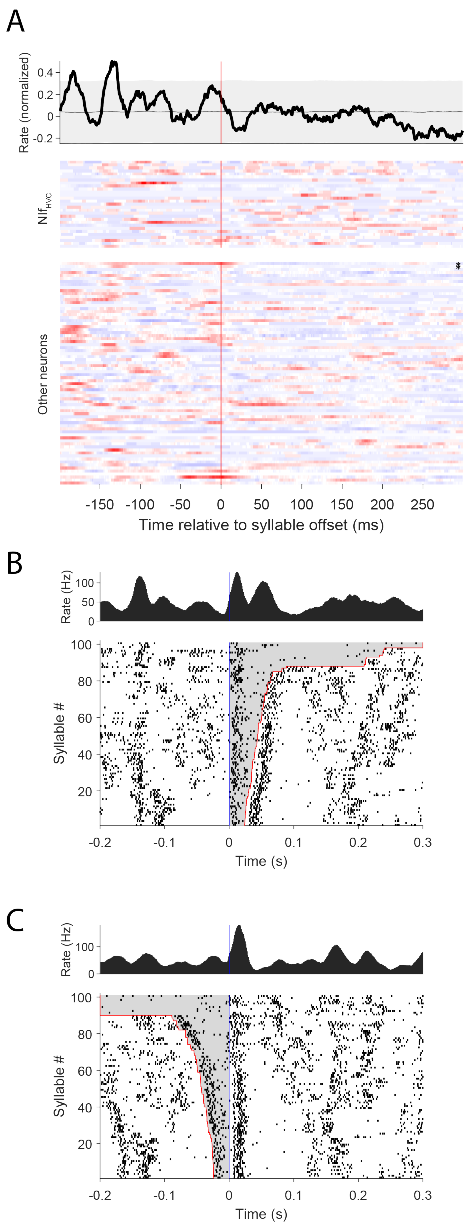

Figure S4: Characterization of NIf activity at syllable offsets

(A) Population summary of NIf activity during tutoring aligned to offsets of the last syllable in a bout of tutor song. The two neurons with a significant offset response are marked by asterisk at right. (B) Syllable-offset aligned raster and PSTH for the neuron with the most significant offset response. Syllable offsets are sorted according to the duration of the following gap, indicated by shaded area. (C) Syllable-onset aligned raster and PSTH for this same neuron in B. Here, syllable onsets are sorted according to the duration of the preceding gap. Note that this neuron responds immediately after both syllable onsets and syllable offsets.

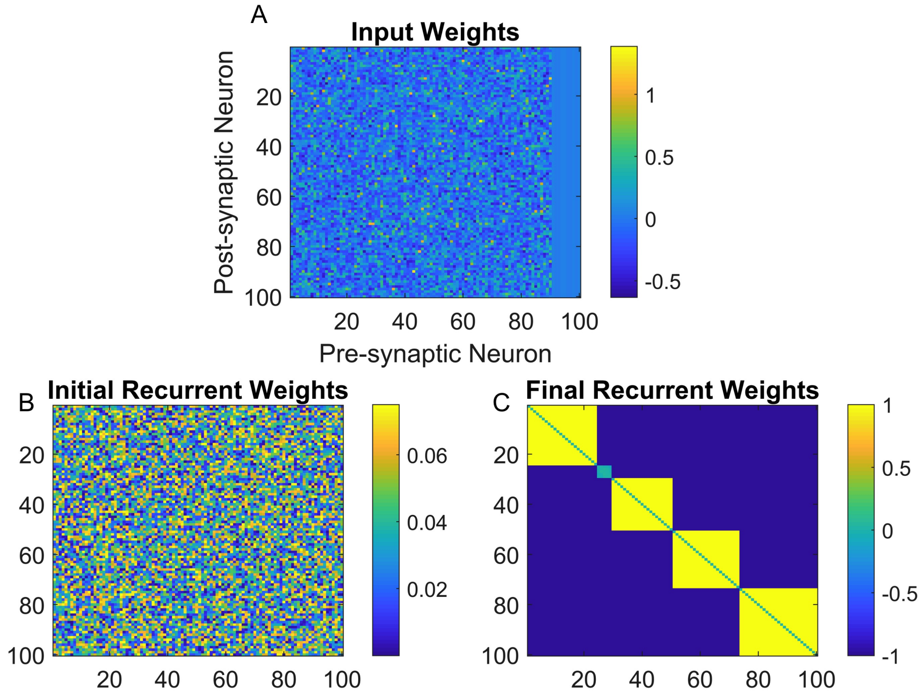

Figure S5: Model weight matrices

(A) Weights from input layer to NIf network. Note that final ten input neurons (last ten columns) have equal weights onto all neurons in the NIf network. These are proposed "onset" neurons. (B) Initial random recurrent weights within the NIf network. (C) Final learned recurrent weights within the NIf network. Note that, as shown in the t-SNE plots in the main figure, 4 large ensembles have been formed.
