# Supplementary material for: An avian cortical circuit for chunking tutor song syllables into simple vocal-motor units
Source: Nat Commun. 2020 Oct 6;11:5029. doi: 10.1038/s41467-020-18732-x (PMC7538968; doi:10.1038/s41467-020-18732-x)
Supplement: Supplementary file 3 — Reporting Summary [file 41467_2020_18732_MOESM3_ESM.pdf]

nature research

Corresponding author(s): Michale Fee

Last updated by author(s): Aug 4, 2020

Reporting Summary

Nature Research wishes to improve the reproducibility of the work that we publish. This form provides structure for consistency and transparency in reporting. For further information on Nature Research policies, see Authors & Referees and the Editorial Policy Checklist.

Statistics

For all statistical analyses, confirm that the following items are present in the figure legend, table legend, main text, or Methods section.

n/a	Confirmed	
☐	☒	The exact sample size (n) for each experimental group/condition, given as a discrete number and unit of measurement
☐	☒	A statement on whether measurements were taken from distinct samples or whether the same sample was measured repeatedly
☐	☒	The statistical test(s) used AND whether they are one- or two-sided<br>*Only common tests should be described solely by name; describe more complex techniques in the Methods section.*
☒	☐	A description of all covariates tested
☐	☒	A description of any assumptions or corrections, such as tests of normality and adjustment for multiple comparisons
☐	☒	A full description of the statistical parameters including central tendency (e.g. means) or other basic estimates (e.g. regression coefficient) AND variation (e.g. standard deviation) or associated estimates of uncertainty (e.g. confidence intervals)
☐	☒	For null hypothesis testing, the test statistic (e.g. F, t, r) with confidence intervals, effect sizes, degrees of freedom and P value noted<br>*Give P values as exact values whenever suitable.*
☒	☐	For Bayesian analysis, information on the choice of priors and Markov chain Monte Carlo settings
☒	☐	For hierarchical and complex designs, identification of the appropriate level for tests and full reporting of outcomes
☐	☒	Estimates of effect sizes (e.g. Cohen's d, Pearson's r), indicating how they were calculated

Our web collection on statistics for biologists contains articles on many of the points above.

Software and code

Policy information about availability of computer code

Data collection	Data was collected using previously described MATLAB software (MATLAB version 2007a).
Data analysis	Data was analyzed using custom MATLAB software (MATLAB version R2018b). Analysis code and code to generate the data figures is posted alongside the raw data on the CRCNS data sharing platform, doi: http://dx.doi.org/10.6080/K0GQ6W0K . MATLAB code to generate the model is provided as supplemental information (tested on MATLAB versions 2018a and 2017a). The model movie was generated using the dynamic t-SNE algorithm (https://github.com/paulorauber/thesne, version: initial commit).

For manuscripts utilizing custom algorithms or software that are central to the research but not yet described in published literature, software must be made available to editors/reviewers. We strongly encourage code deposition in a community repository (e.g. GitHub). See the Nature Research guidelines for submitting code & software for further information.

Data

Policy information about availability of data

All manuscripts must include a data availability statement. This statement should provide the following information, where applicable:

- Accession codes, unique identifiers, or web links for publicly available datasets
- A list of figures that have associated raw data
- A description of any restrictions on data availability

Data (raw electrophysiology and audio data, metadata, and annotations), as well as MATLAB code to generate data figures, is publicly available on CRCNS (doi: http://dx.doi.org/10.6080/K0GQ6W0K). MATLAB code to run the model and generate figures related to the model is provided as supplementary material.

Field-specific reporting

Please select the one below that is the best fit for your research. If you are not sure, read the appropriate sections before making your selection.

☒ Life sciences ☐ Behavioural & social sciences ☐ Ecological, evolutionary & environmental sciences

For a reference copy of the document with all sections, see nature.com/documents/nr-reporting-summary-flat.pdf

Life sciences study design

All studies must disclose on these points even when the disclosure is negative.

Sample size	Sample size was chosen to be similar to sample size in similar types of studies. (Vyssotski et al. 2016, Hahnloser et al. 2002)
Data exclusions	No data were excluded from the analysis.
Replication	The experiments were not explicitly replicated as an independent hypothesis test. However, robust syllable onsets responses were observed during singing and tutoring in most birds recorded. Of the 13 birds in which NIf neurons were recorded during singing, 11 had song-locked neurons. The two birds in which singing-locked neurons were not observed had only small numbers of neurons recorded (5 and 4 total NIf neurons recorded during singing respectively). Of the 14 birds in which NIf neurons were recorded during tutoring, 11 had tutor-locked neurons. The three birds in which tutor-locked neurons were not observed had only one or two total NIf neurons recorded during tutoring.
Randomization	N/A (samples were not allocated into to groups, since we were not studying the effects of an experimental manipulation)
Blinding	N/A (samples were not allocated into to groups, since we were not studying the effects of an experimental manipulation)

Reporting for specific materials, systems and methods

We require information from authors about some types of materials, experimental systems and methods used in many studies. Here, indicate whether each material, system or method listed is relevant to your study. If you are not sure if a list item applies to your research, read the appropriate section before selecting a response.

Materials & experimental systems

n/a	Involved in the study	
☒	☐	Antibodies
☒	☐	Eukaryotic cell lines
☒	☐	Palaeontology
☐	☒	Animals and other organisms
☒	☐	Human research participants
☒	☐	Clinical data

Methods

n/a	Involved in the study	
☒	☐	ChIP-seq
☒	☐	Flow cytometry
☒	☐	MRI-based neuroimaging

Animals and other organisms

Policy information about studies involving animals; ARRIVE guidelines recommended for reporting animal research

Laboratory animals	Zebra finch (Taeniopygia guttata), juvenile (40-90dph) males
Wild animals	No wild animals were used in the study.
Field-collected samples	No field collected samples were used in the study.
Ethics oversight	Animal care and experiments were carried out in accordance with NIH guidelines, and reviewed and approved by the Massachusetts Institute of Technology Committee on Animal Care.

Note that full information on the approval of the study protocol must also be provided in the manuscript.

October 2018
