# Supplementary material for: An avian cortical circuit for chunking tutor song syllables into simple vocal-motor units
Source: Nat Commun. 2020 Oct 6;11:5029. doi: 10.1038/s41467-020-18732-x (PMC7538968; doi:10.1038/s41467-020-18732-x)
Supplement: Supplementary file 4 — Description of Additional Supplementary Files [file 41467_2020_18732_MOESM4_ESM.pdf]

Description of Additional Supplementary Files

File Name: Supplementary Code 1
Description: MATLAB code for the NIf model and the combined NIf/HVC model. For instructions on how to reproduce any of the models in the manuscript, refer to the README included with the code.

File Name: Supplementary Movie 1
Description: This movie illustrates the network evolution over time. White circles represent neurons, red lines represent strong inhibitory weights, blue lines represent strong excitatory weights and gray lines represent weak weights. Positions of neurons are determined by a t-SNE clustering approach carried out on the network's weight matrix at each timestep.

Initially, there is little structure in the network (as in Figure 7C), but neural ensembles slowly form. In one ensemble at a time, recurrent weights become very strong, and inhibitory weights build up between the ensemble and the rest of the network (as in Figure 7D). The final state of the network is four recurrently-connected ensembles that inhibit each other (as in Figure 7E). A small number of neurons (in the center of the clustering) fail to join any ensemble due to relatively weaker recurrent weights.
