# Supplementary material for: An avian cortical circuit for chunking tutor song syllables into simple vocal-motor units
Source: Nat Commun. 2020 Oct 6;11:5029. doi: 10.1038/s41467-020-18732-x (PMC7538968; doi:10.1038/s41467-020-18732-x)
Supplement: Supplementary file 5 — Supplementary Code 1 [file 41467_2020_18732_MOESM5_ESM.zip › NIf Paper - code to publish - Sept2020/README.rtf]

Michael Happ, 2020~~~These folders contain MATLAB code for different variations of the NIf model and NIf/HVC model used in the paper, “An avian cortical circuit for chunking tutor song syllables into simple vocal-motor units.”The folder “Vanilla NIf Model” contains the most basic version of the NIf model, used in Figure 7. Important scripts include NIf_mainScript, which actually runs the model when executed. Figures are generated, too.The folder “NIf to HVC Model” contains scripts for running the combined NIf-HVC model, as used in Figure 8. Note that there is not a single script that runs the entire combined model. Rather, the combined model is run by generating a NIf output and using that to drive and HVC model. The proper way to run the Nif to HVC model is to first run NIf_mainScript_outputAltDiff (or any other version of the NIf model that outputs A, ordering and singTS. Next one should run nifToHVC_process, which processes the NIf output to be fed into the HVC model. Finally, run AlternatingDifferentiation_nifHVC. It should be noted that this folder already contains processed NIf output data, so figures can be recreated by simply running AlternatingDifferentiation_nifHVC.The folders “Rhythmic HVC Stimulation” and “Sporadic HVC Stimulations” are used to generate the alternate-timing versions of the model used in Figure 8. The script to execute for figure generation are “hvc_RhythmicStim” in the former and “HVCModel_sporadicSims” in the latter.
